# Study on the Preparation and Process Parameter-Mechanical Property Relationships of Carbon Fiber Fabric Reinforced Poly(Ether Ether Ketone) Thermoplastic Composites

**DOI:** 10.3390/polym16070897

**Published:** 2024-03-25

**Authors:** Yan Wang, Yanchao Yang, Hongbo Zhang, Siwen Ding, Ting Yang, Jinhui Pang, Haibo Zhang, Jinling Zhang, Yunhe Zhang, Zhenhua Jiang

**Affiliations:** Key Laboratory of High Performance Plastics, Ministry of Education, National & Local Joint Engineering Laboratory for Synthesis Technology of High Performance Polymers, College of Chemistry, Jilin University, Changchun 130012, China; wyan2012@jlu.edu.cn (Y.W.); yangyanchao@jlu.edu.cn (Y.Y.); zhanghb20@mails.jlu.edu.cn (H.Z.); dingsw23@mails.jlu.edu.cn (S.D.); yangting1988@jlu.edu.cn (T.Y.); pangjinhui@jlu.edu.cn (J.P.); zhanghaib@jlu.edu.cn (H.Z.); jiangzhenhua@jlu.edu.cn (Z.J.)

**Keywords:** Carbon fiber fabric-reinforced poly(ether ether ketone), processing parameters, mechanical properties, thermoplastics

## Abstract

Carbon fiber fabric-reinforced poly(ether ether ketone) (CFF-PEEK) composites exhibit exceptional mechanical properties, and their flexibility and conformability make them a promising alternative to traditional prepregs. However, the formation of the CFF-PEEK composite is trapped in the high viscosity of PEEK, the smooth surface, and tightly interwoven bundles of CFF. It is more difficult for the resin to flow through the fibers of complex textile structures. Here, a simple film stacking method using the hot-pressing process of plain-woven CFF-PEEK thermoplastic composites is discussed. The uniform distribution of PEEK resin between each layer of CFF reduces the flow distance during the molding process, preventing defects in the composite material effectively. Four process parameters, including molding temperature (370, 385, 400, and 415 °C), molding pressure (1, 2, 4, 8, and 10 MPa), molding time (10, 20, 30, 40, 60, and 90 min), and pre-compaction process, are considered. Interlaminar shear strength (ILSS), tensile strength, and flexural strength of CFF/PEEK composites are evaluated to optimize the process parameters. Moreover, ultrasonic scanning microscopy and scanning electron microscopy are employed to observe the formation quality and microscopic failure modes of CFF/PEEK composites, respectively. The ultimate process parameters are a molding temperature of 415 °C, molding pressure of 10 MPa, molding time of 60 min, and the need for the pre-compaction process. Under the best process parameters, the ILSS is 62.5 MPa, the flexural strength is 754.4 MPa, and the tensile strength is 796.1 MPa. This work provides valuable insight for studying the process parameters of fiber fabric-reinforced thermoplastic polymer composites and revealing their impact on mechanical properties.

## 1. Introduction

Nowadays, carbon fiber (CF)-reinforced resin composites are extensively applied as structural materials in the fields of aviation, aerospace, automotive industries, and civil high-technology owing to their high strength, stiffness, and processibility [1,2]. CF-reinforced resin composites can be generally classified into CF-reinforced thermoset polymer composites and CF-reinforced thermoplastic polymer composites according to the nature of the resin [3]. However, with the increasing demand for high-performance composites in industries and the requirement for sustainable development of industrial products, CF-reinforced thermoset polymer composites have difficulty meeting the requirements due to their irreversible curing reaction. It is impossible to remelt and remold them after reaching the end of their service life, and it is also difficult to recycle and reuse them [4,5]. CF-reinforced thermoplastic polymer composites have attracted more and more attention because of their excellent damage resistance and recyclability [6]. As an excellent representative thermoplastic composite, CF reinforced poly(ether ether ketone) (CF/PEEK) composites are attracting increasing attention owing to their chemical resistance, heat resistance, remarkable mechanical properties, and good wear resistance [3,7,8]. When subjected to external forces, the load carried by CF/PEEK composites is transferred from the matrix to the reinforcing carbon fibers [9]. The mechanical properties of the composites are influenced by the ability of the interface to transfer stress, and the ability of the interface to transfer stress is closely related to the bonding between the fibers and the matrix [10]. This can be described by interfacial shear strength (IFSS), but IFSS only reflects the bonding between fibers and resin at a microscopic level and does not take into account the mutual influence of fibers around the fiber/resin interface, as well as complex residual stresses that may be produced in samples during the production process [11,12].

Recently, various methods have been employed in fabricating CF/PEEK composites, including the melt impregnation method, fiber commingled method, and powder impregnation method [13,14]. However, on account of the high melting viscosity, poor fluidity, high melting point, and insolubility in many solvents of PEEK, as well as the smooth surface of CF, it is extremely challenging to disperse the molten PEEK resin in the CF bundles as uniformly as possible, and tiny spaces between fibers cannot be impregnated well by PEEK easily [15,16]. How to make molten PEEK resin sufficiently able to impregnate the CF bundles is a scientific problem that needs to be solved. The film stacking method is an enhanced approach to the melt impregnation method, which involves the application of a uniform layer of thermoplastic resin film combined with reinforced fiber or fabric, followed by hot-pressing to achieve melting and immersion [2]. In terms of the structural design of its composites, 2D woven carbon fiber fabric (CFF) can better balance the mechanical responses in the orthogonal directions, so the orthogonal plain-woven CFF/PEEK composites have broad application prospects in many fields such as aerospace, automotive industry, and others [17,18]. The film stacking method of PEEK film and CFF can solve the difficulty of extending molten PEEK in the plane direction [19,20]. However, owing to the tightly interwoven bundles of CFF, it is more challenging for molten resin to flow through the fibers of these complex textile structures. Making the resin infiltrate into the surface of a single CF better requires selecting an appropriate hot-press molding process [14,21,22]. More importantly, the hot-press molding process is to make raw materials into the required composite products, and the hot-press molding process parameters have an extraordinary influence on determining the interfacial bonding between matrix and fibers, forming quality, and comprehensive mechanical properties [23,24]. Sufficiently high pressures and molding temperatures are required to make PEEK impregnate the CFF bundles uniformly and to obtain structurally dense fiber-reinforced thermoplastic composites. Hence, it is exceedingly noteworthy and essential to study the relationships between the parameters, composites, structure, and mechanical properties of CFF/PEEK composites during the hot-press molding process and reveal the internal influence mechanism.

Herein, CFF/PEEK-laminated composites are fabricated using a simple film stacking method of PEEK film and CFF. The influence of hot-press molding process parameters, including temperature, pressure, time, and pre-compaction process, on composites was studied. The rheological behavior was studied using a capillary rheometer to reveal the melting viscosity of PEEK. Ultrasonic testing was employed to detect internal flaws or defects in the laminates. The mechanical properties and thermal properties of composites were also studied. The mechanism of the molding parameters on the mechanical properties of CFF/PEEK composites was revealed by observing tensile specimens of composites with scanning electron microscopy.

## 2. Materials and Methods

### 2.1. Materials and Reagents

CFF (J9-2019066-1 K) was purchased from Jilin Jiyan High-tech Fiber Co., Ltd., Jilin, China. The warp tensile strength and weft tensile strength of CFF were 1655.2 N/25 mm and 1587.30 N/25 mm, respectively. PEEK films (with a thickness of 70 μm) were supplied by Jilin University Special Plastic Engineering Research Co., Ltd., Changchun, China. The melt index of PEEK film was 27 g/10 min, T*_g_* was 145 °C, Tm was 338 °C, and tensile strength was 76.5 MPa. Ethanol was purchased from Xilong Chemical Industry Group Co., Ltd., Shantou, China. and used as received.

### 2.2. Characterization

#### 2.2.1. Thermal Analyses

The thermal properties of PEEK film were characterized to know the thermophysical properties of PEEK resin in a high-temperature molten state. Thermogravimetric analysis (TGA) of PEEK film was performed by TGA/DSC 1 (Mettler-Toledo., Ltd., Zurich, Switzerland) to investigate the thermal stability of PEEK resin under a high-temperature environment. The PEEK film was cut, and the quality of each sample of TGA was about 10 mg. The heating procedure of TGA was as follows: the sample was heated from 100 °C to 800 °C at a heating rate of 10 °C/min in air and N_2_ environment, respectively. The gas flow rate was 50 mL/min. Differential scanning calorimetry (DSC) analysis was carried out using DSC821e (Mettler-Toledo., Ltd., Zurich, Switzerland) to observe the glass transition temperature (T*_g_*) and melting temperature (T*_m_*) of PEEK. The PEEK film was cut, and the quality of each sample of DSC was about 3–10 mg. The heating procedure of DSC was as follows: the sample was heated from 25 °C to 400 °C at a heating rate of 10 °C/min and the gas flow rate of 50 mL/min.

#### 2.2.2. Rheological Analysis

A capillary rheometer (Rheograph 25, GÖTTFERT Werkstoff-Prüfmaschinen GmbH, Buchen, Germany), with a Φ 20.0 mm parallel disk and 1.0 mm gap, was used to evaluate the shear viscosity of the PEEK films at different molten temperatures (370 °C, 385 °C, 400 °C, and 415 °C). The shear rate was set to 50–5700 s^−1^.

#### 2.2.3. Microstructure Analysis

The microscopic morphology was observed with a scanning electron microscope (Nova NanoSEM 450: FEI., Thermo Fisher Scientific Inc., Waltham, MA, USA). The test sample was attached to the sample table with conductive adhesive and then coated with platinum in a high vacuum mode for testing.

#### 2.2.4. Ultrasonic Analysis

Ultrasonic testing is a non-destructive testing method that uses high-frequency sound waves to detect internal flaws or defects in materials. In the case of composite materials, ultrasonic testing can be used to examine their internal microstructure, defects, and damage characteristics. The internal microstructure, defects, and damage characteristics of the CFF/PEEK composites were observed with a scanning acoustic microscope (PVA SAM 300: PVA TePla Analytical Systems GmbH, Westhausen, Germany). The sample was placed in deionized water, and a transducer with a frequency of 30 MHz was used to generate sound waves that were transmitted through the material. The reflected waves were analyzed to create an image of the internal structure of the material.

#### 2.2.5. Mechanical Properties Test

The mechanical properties of CFF/PEEK composites were tested using the electronic universal material testing machine AGX (AGX: Shimadzu Scientific Instruments, Kyoto, Japan). Unless otherwise explained, all mechanical properties tests were carried out in the standard laboratory atmosphere (100 KN, 23 °C, and 50% relative humidity). The ILSS was measured by using the short-beam shear (SBS) test according to ASTM-D2344’s [25] procedures. The short-beam shear specimen was cut to a specimen size of 30 mm × 10 mm × 3 mm, the crosshead rate was 1 mm/min, and the ratio of span to thickness was 5:1. The bending test specimens were cut to a specimen size of 120 mm × 12.5 mm × 3 mm. The ratio of span to thickness was selected to be 32:1. The crosshead rate was 5 mm/min for measuring flexural strength and 1 mm/min for measuring flexural modulus. The tensile properties were measured with reference to ASTM-D3039’s [26] procedures; the crosshead rate was 1 mm/min, and the extensometer length was 50 mm. The tensile test specimens were cut to a specimen size of 150 mm × 12.5 mm × 1.5 mm. Glass fiber-reinforced epoxy resin plates were used as reinforcement sheets. The flexural properties were measured with reference to ASTM-D7264’s [27] procedures. All mechanical properties were tested at least five times.

### 2.3. Preparation of CFF/PEEK Composites

**Figure 1:**
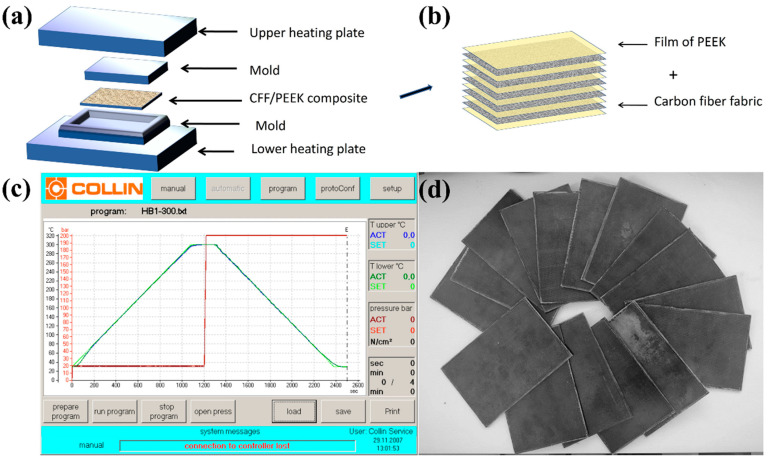
Manufacturing of CFF/PEEK composite: (**a**,**b**) schematic of fabrication process; (**c**) molding procedure; and (**d**) picture of CFF/PEEK composites laminate.

In this work, the hot-press method was used to prepare the CFF/PEEK composites. Firstly, the PEEK film (10 cm × 15 cm) was washed with ethanol and vacuum-dried at 60 °C for 4 h. Then, the carbon fiber fabric and PEEK film were placed in the mold layer by layer, as shown in Figure 1a. The mold was put into the vacuum hot-press machine (Model: P 400PV, COLLIN, Maitenbeth, Germany), as shown in Figure 1b. Next, the machine was set with the program shown in Figure 1c, which changed the temperature, pressure, time, and other molding conditions for hot-pressing. After the program, the mold cooled to room temperature. Finally, the internal and external pressure of the chamber was balanced, and the mold was brought out. The CFF/PEEK-laminated composite was obtained after molding (Figure 1d).

## 3. Results

### 3.1. Thermal Behavior of PEEK

The thermophysical properties of PEEK resin, including T*_g_*, T*_m_*, decomposition temperature, and melt viscosity, are of great significance for designing CFF/PEEK composites [28,29]. Hence, it is necessary to characterize the thermal and physical properties of PEEK films first before exploring the molding process parameters of CFF/PEEK composites.

**Figure 2:**
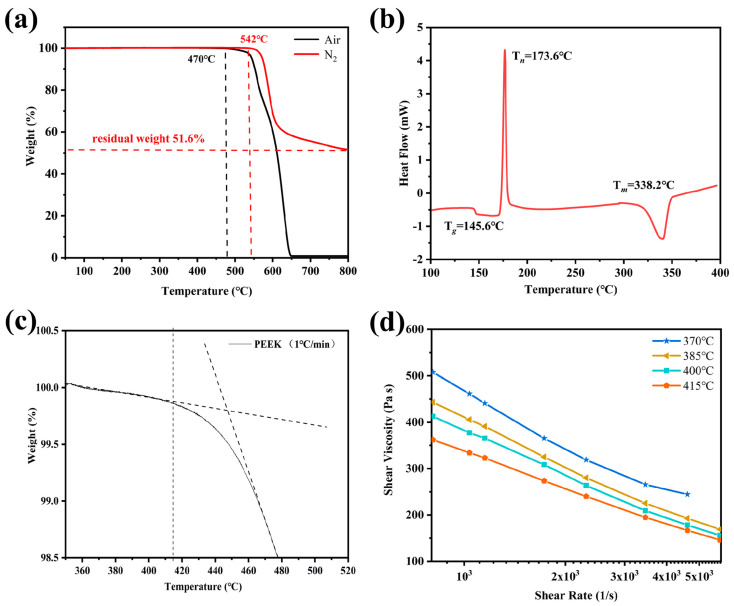
The thermophysical properties of PEEK film: (**a**) TGA curves; (**b**) DSC curve; (**c**) TGA curve (a heating rate of 1 °C/min); and (**d**) viscosity versus shear rate at different temperatures.

The thermal stability of PEEK resin is essential for the process and service of CFF/PEEK composites. Thus, TGA was used for exploring the thermal stability of PEEK. While the thermal stability of PEEK in an oxygen-containing and oxygen-free atmosphere is quite different, the TGA of PEEK in air and N_2_ environments were employed, respectively (Figure 2a) [30]. In the air environment, the decomposition onset temperature of PEEK is about 470 °C. When the temperature rises to 544 °C, the weight loss of PEEK sharply increases, indicating severe thermal decomposition of PEEK, mainly due to ether bond and ketone bond breakage [31]. With temperature further increasing, the thermal decomposition of PEEK continues. When the temperature reaches 650 °C, O_2_ finally oxidizes the PEEK into coke. In contrast, the decomposition onset temperature of PEEK is delayed to 542 °C in a nitrogen atmosphere. Furthermore, the thermal weight loss of PEEK is only 50% when the temperature increases to 800 °C. This allows the use of vacuum press machines to reduce contact with oxygen during the composite material preparation process.

Figure 2b shows the DSC curve of PEEK film with T*_g_* at 145.6 °C, cold crystallization temperature (T*_n_*) at 173.6 °C, and T*_m_* at 338.2 °C, respectively. The DSC curve reflects transition temperatures from a hard glass state to a highly elastic state and then a viscous flow state, which directly shows polymer chain mobility ability. Therefore, the hot-press molding process temperature used in this work should not be lower than 350 °C to ensure the resin reaches a flow state.

In order to explore the upper limit processing temperature, a thermal degradation test was conducted by heating PEEK film at a heating rate of 1 °C/min from 350 °C to 500 °C, as shown in Figure 2c. The slight degradation of PEEK film at high temperatures indicates its instability under prolonged exposure to high temperatures, which limits upper limit processing temperature.

The melt viscosity serves as a quantitative measure of flow resistance, providing crucial information for the processing of CFF/PEEK composites by directly reflecting polymer fluidity in relation to temperature [32]. The wettability of PEEK resin with CFF and the bonding strength between them are directly influenced by the fluidity of PEEK resin. Therefore, a capillary rheometer was employed to measure the viscosity of PEEK at different temperatures. As depicted in Figure 2d, an increase in temperature resulted in a decrease in the viscosity of PEEK resin. This phenomenon can be attributed to an expansion in the free volume within the PEEK melt as well as an acceleration in chain segment mobility, leading to weakened intermolecular interaction forces and ultimately promoting the fluidity of PEEK melt.

### 3.2. Effect of Hot-Pressing Temperature on the Properties of CFF/PEEK Composite

**Figure 3:**
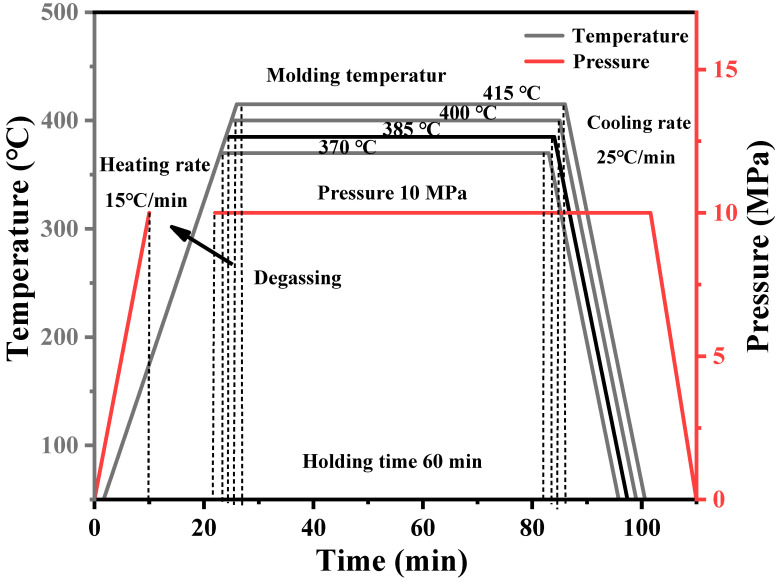
Molding procedure of CFF/PEEK composites with different temperatures.

The processing technology of composite materials has a great influence on the structure and properties of composite materials. Thus, the compression molding process of composite materials is the technical basis and core for preparing high-performance composite. One of the important process parameters in compression is hot-pressing temperature. Since CFF can withstand temperatures above 500 °C, the temperature in the hot-pressing process is determined by the thermoplastic resin matrix. According to the melt viscosity of PEEK, when hot-pressing PEEK resin and CFF, the lower the melt viscosity of the resin, the easier it is to infiltrate the CFF, allowing it to form a good interface. From this perspective, the higher the hot-pressing temperature, the more the interface between PEEK and CFF becomes tighter. However, due to the high melting point of PEEK resin (334 °C), the hot-pressing temperature generally needs to be above 370 °C or even 400 °C. During the hot-pressing process, the PEEK resin is exposed to such a high-temperature environment for a long time, various side reactions, such as degradation reaction, crosslinking reaction, oxidation reaction, etc., will take place. The side reactions would seriously affect the structure and performance of the resin, resulting in a decrease in the performance of the final composite. Therefore, controlling the hot-pressing temperature during the entire processing process is particularly important for affecting the mechanical properties of the CFF/PEEK composite. Four hot-pressing temperatures of 370 °C, 385 °C, 400 °C, and 415 °C were considered to press carbon fiber fabric/PEEK composite material, and the compression molding process is shown in Figure 3.

**Figure 4:**
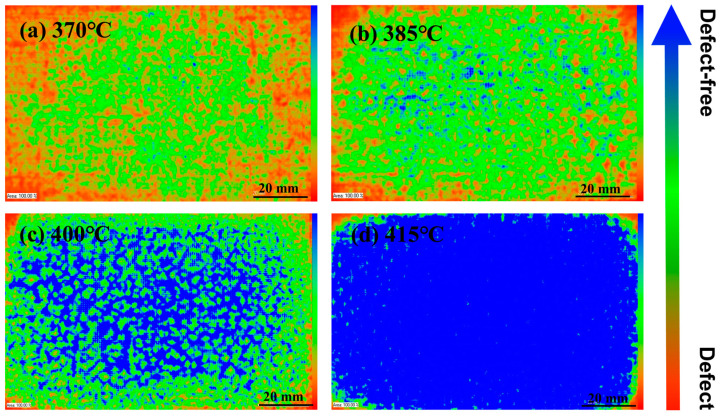
C-SAM images of CFF/PEEK composites with different molding temperatures.

The internal structure of CFF/PEEK composite was investigated using C-mode scanning acoustic microscopy (C-SAM). The blue part in the image represents the fully transmitted part where the ultrasonic wave propagates well, which is the defect-free region. The green part represents the area where the ultrasonic wave propagates poorly, and the red part is usually regarded as defects due to holes caused by insufficient compaction inside the product. As shown in Figure 4a, it can be seen that when the hot-pressing temperature is 370 °C, the composite material has larger holes and defects inside. This is because at lower temperatures, the PEEK resin has high viscosity and poor fluidity, and the molten resin cannot infiltrate into the grooves of the carbon fiber fabric, resulting in inadequate impregnation of the fibers. From Figure 4b–d, as the hot-pressing temperature increases, the blue region gradually increases, and the defect decreases. With the increase in hot-pressing temperature, the resin’s fluidity improves, allowing it to infiltrate into the grooves of the carbon fiber. The increasing hot-pressing temperature can enhance the bonding performance between PEEK resin and carbon fiber fabric, reducing defects inside the composite material.

**Figure 5:**
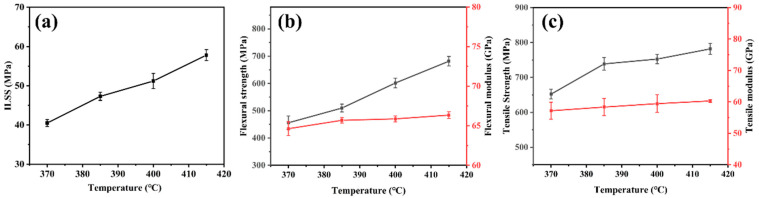
Relationships between mechanical properties and molding temperature of CFF/PEEK composites: (**a**) ILSS; (**b**) flexural strength and flexural modulus; and (**c**) tensile strength and tensile modulus.

Table 1 shows the mechanical properties of CFF/PEEK composites under different hot-pressing temperatures. Figure 5 shows the effect of hot-pressing temperature on the mechanical properties of composites. From the figure, within the studied range, as the hot-pressing temperature increases from 370 °C to 415 °C, the ILSS of the composite gradually increases, increasing by 43% from 40.5 MPa to 57.8 MPa. The flexural strength and tensile strength also show a linear increase trend, reaching their highest strength at 415 °C. The flexural strength increased by 42%, from 455.9 MPa to 681.5 MPa, while the tensile strength increased by 20%, increasing from 652.2 MPa to 781.8 MPa. This is mainly due to the fact that the fluidity of PEEK resin increases with temperature increase. When the molding temperature is low, the viscosity of PEEK resin melt is high. The high viscosity of melt PEEK resin makes it difficult to penetrate into the warp and weft yarns of the CFF, resulting in poor interfacial bonding between the resin and fibers. Under external force, it is easier to cause delamination failure and cannot effectively bear stress. With increasing temperature, the wettability of the resin to the CFF improves as well, and the interfacial bonding performance between the two phases becomes stronger. When subjected to stress, the base resin PEEK can better transmit and distribute stress to the fibers, thereby improving the overall mechanical properties of the composite material. Therefore, within the studied temperature range, a hot-pressing temperature of 415 °C was selected.

**Figure 6:**
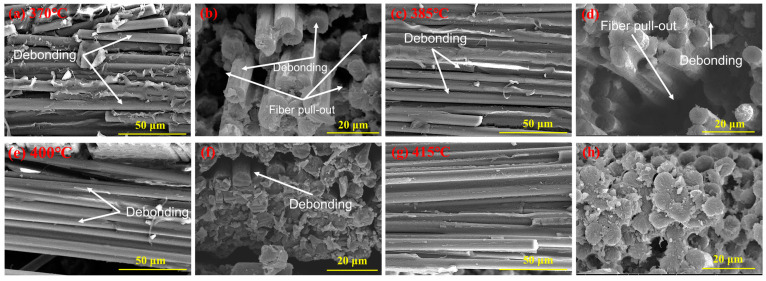
SEM images of typical fracture surfaces of CFF/PEEK composites by different molding temperatures: (**a**,**b**) 370 °C; (**c**,**d**) 385 °C; (**e**,**f**) 400 °C; and (**g**,**h**) 415 °C.

To further reveal the mechanism of the internal influence of molding temperature on the mechanical properties of CFF/PEEK composites, the cross-sectional morphologies (including perpendicular and parallel to the CFF direction) of tensile specimens of CFF/PEEK composites were observed with scanning electron microscopy (Figure 6). When the molding temperatures were 370 °C, 385 °C, and 400 °C (Figure 6a–f), voids caused by fiber pullout and fiber–matrix debonding were observed obviously. This can be attributed to the PEEK matrix having a higher viscosity at lower molding temperatures. The molten PEEK resin does not easily flow between the fibers, resulting in obvious cracks and resin-free areas in the composites, which leads to a poor infiltration level of the resin into the fibers. When the molding temperature rises to 415 °C, the samples show step-like fractures, and the cross-section is uniformly wrapped with PEEK resin (Figure 6g,h). This can be explained by the fact that the viscosity of the resin is lowest at 415 °C, which makes the molten PEEK resin easier to penetrate into the fiber bundles. Meanwhile, due to the good penetration performance of the resin matrix, the gaps between the fiber bundles were reduced. This indicates that the molten resin has successfully penetrated into the carbon fiber bundles and enabled them to have good binding ability to resist the destruction brought by external forces.

### 3.3. Effect of Hot-Pressing Pressure on the Mechanical Properties of CFF/PEEK Composite

**Figure 7:**
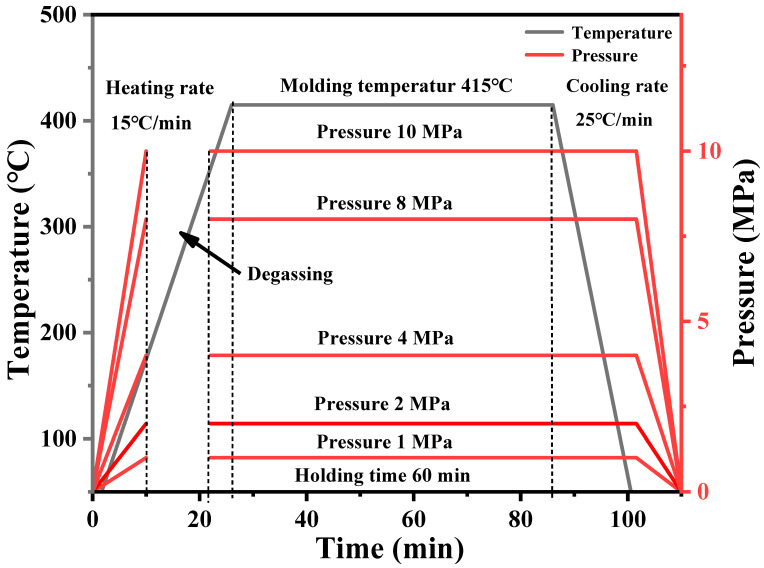
Molding procedure of CFF/PEEK composites with different pressures.

In the hot-pressing molding process, applying pressure is an essential experimental process. The application of pressure allows the PEEK resin to infiltrate into the grooves of the CFF during the melting process, resulting in a carbon fiber-reinforced thermoplastic resin matrix composite. There are large amounts of voids between the layers of PEEK film and CFF. Under a certain pressure, the voids between the layers are compressed, and the air between the layers is also discharged, making the fibers more tightly together. The effect of hot-pressing pressure on the performance of PEEK film-based carbon fiber composite was studied. The size of the pressurized block on the mold is 10 × 15 cm, and the actual pressure applied to the sample is selected as 1, 2, 4, 8, and 10 MPa through calculation. The molding process is shown in Figure 7.

**Figure 8:**
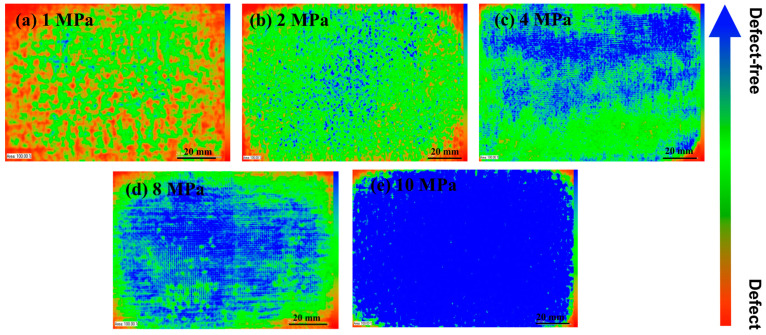
C-SAM images of CFF/PEEK composites with different molding pressures.

Figure 8 shows the C-SAM images of CFF/PEEK composites molded under different pressures. It can be observed from the images that with the increase of hot-pressing pressure, the blue area inside the composite gradually increases, and the defects gradually decrease. Due to its high viscosity, PEEK resin is difficult to flow at low pressure, which makes it impossible to fully infiltrate the carbon fiber fabric, resulting in defects. However, the increasing pressure allows the molten resin to infiltrate the carbon fiber fabric quickly, making the fibers tightly bonded together and reducing defects in the composite.

**Figure 9:**
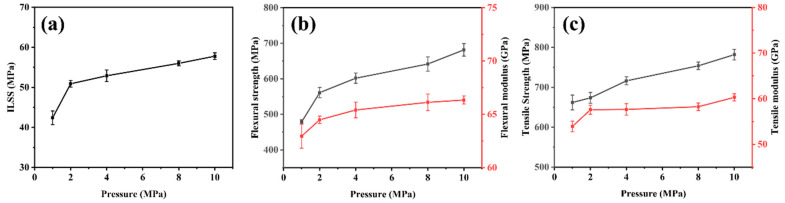
Relationships between mechanical properties and molding pressure of CFF/PEEK composites: (**a**) ILSS; (**b**) flexural strength and flexural modulus; and (**c**) tensile strength and tensile modulus.

Table 2 shows the mechanical properties of CFF/PEEK composites under different hot-pressing pressures. Figure 9 illustrates the effect of hot-pressing pressure on the mechanical properties of the composite material. It can be observed that within the studied range, the shear strength, flexural strength, and tensile strength all show an increasing trend with the increase in hot-pressing pressure. When the hot-pressing pressure is 10 MPa, the mechanical properties of CFF/PEEK composites reach their best. With the increase in molding pressure, the resin can better infiltrate the carbon fiber fabric under higher pressure, and the fibers bond more tightly together, resulting in fewer voids in the composite material and improved mechanical properties. Therefore, 10 MPa was selected as the hot-pressing press within the studied press range.

**Figure 10:**
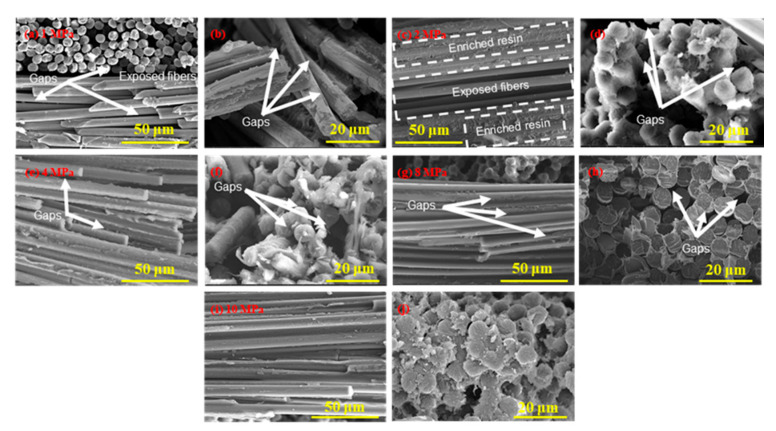
SEM images of typical fracture surfaces of CFF/PEEK composites by different molding pressures: (**a**,**b**) 1 MPa; (**c**,**d**) 2 MPa; (**e**,**f**) 4 MPa; (**g**,**h**) 8 Mpa; and (**i**,**j**) 10 MPa.

The SEM images of fracture morphology from tensile of CFF/PEEK composites under different forming pressures are depicted in Figure 10. At a molding pressure of 1 MPa, numerous exposed fibers and noticeable gaps can be observed (Figure 10a,b), indicating that insufficient molding pressure fails to facilitate smooth infiltration of PEEK resin into CF bundles. When the molding pressure is 2 MPa, many gaps between fibers, fiber–matrix debonding, and enriched resin areas can be seen clearly (Figure 10c,d). This phenomenon may be attributed to insufficient pressure for complete infiltration into the interior of fiber bundles, resulting in resin accumulation on the surface. As the pressure rises to 4 MPa and 8 MPa (Figure 10e–h), holds within the composite material gradually decrease while achieving a more uniform distribution of resin within fiber bundles. However, some defects and voids still remain. When the molding pressure is 10 MPa, the fracture surface appears relatively flat without fiber pullout or fiber–matrix debonding, as shown in Figure 10i,j. This also means the molten PEEK resin can impregnate the fiber very well.

### 3.4. Effect of Hot-Pressing Time on the Mechanical Properties of CFF/PEEK Composite

**Figure 11:**
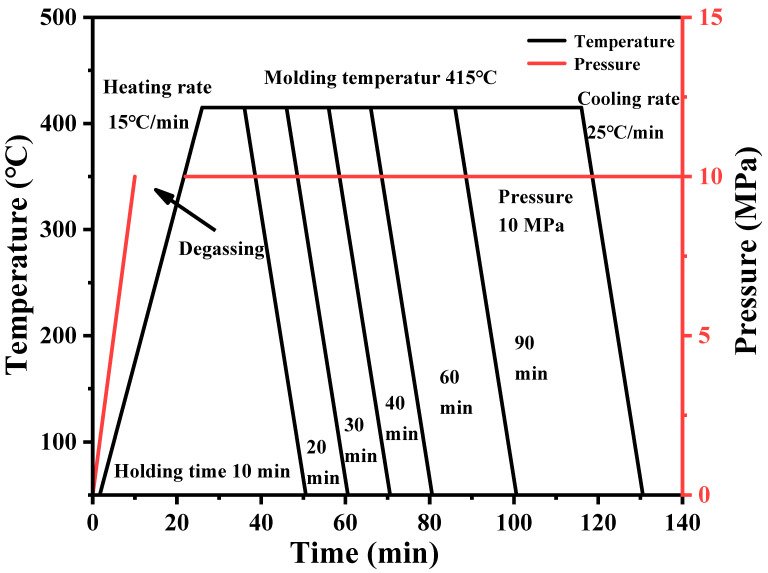
Molding procedure of CFF/PEEK composites with different times.

The effect of hot-pressing time on the mechanical properties of CFF/PEEK composite is significant. A shorter time can improve production efficiency. However, due to the large viscosity of the resin matrix melt and slow flow rate, it will take time for the resin to infiltrate the fiber bundles. Moreover, the large volume of resin and the dense distribution of fibers have a certain obstructive effect on the flow of resin. If the hot-pressing time is too short, the resin liquid may not have enough time to infiltrate the reinforcing materials, and fiber may be deformed due to increased pressure. Therefore, six different hot-pressing times of 10, 20, 30, 40, 60, and 90 min were selected to explore the performance of the composite material. The molding process is shown in Figure 11.

**Figure 12:**
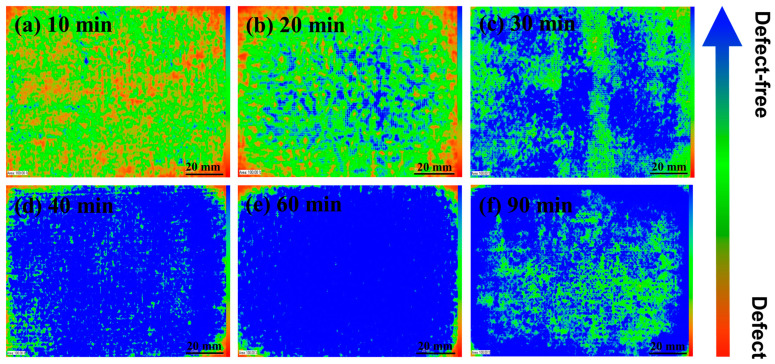
C-SAM images of CFF/PEEK composites with different molding times.

The effect of hot-pressing time on the internal structure of the CFF/PEEK composite is significant. Figure 12 shows the C-SAM images of the composite materials molded under six different hot-pressing times. It can be seen from the images that with the increase of hot-pressing time, the CFF/PEEK composite gradually becomes more compact, and the area of defects significantly decreases. However, when the time increases to 90 min, scattered defects appear in the composite material relatively. This may be owing to the long-term high-temperature and high-pressure conditions causing partial crosslinking of the resin and an increase in viscosity, making it difficult for the CFF to be well impregnated.

**Figure 13:**
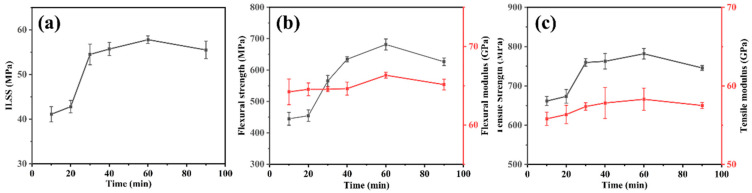
Relationships between mechanical properties and molding time of CFF/PEEK composites: (**a**) ILSS; (**b**) flexural strength and flexural modulus; and (**c**) tensile strength and tensile modulus.

Table 3 shows the mechanical properties of CFF/PEEK composites under different molding time. Figure 13 illustrates the effect of hot-pressing time on the mechanical properties of the composite material. It can be seen from the Figure 3 that the ILSS, flexural, and tensile properties of the CFF/PEEK composites show a trend of first increasing and then decreasing with the extension of hot-pressing time, which is consistent with the results of ultrasonic scanning. The mechanical properties reach their maximum values at a molding time of 60 min, with a ILSS of 57.8 MPa, a flexural strength of 681.5 MPa, and a tensile strength of 781.8 MPa. This is because that within the range of 10 min to 60 min, the extension of constant temperature time helps the resin to flow better and infiltrate the CFF, thus improving the mechanical properties of the composite. However, with the increased molding time, the resin in a long-term high-temperature and high-pressure environment undergoes crosslinking, degradation, and oxidation, resulting in a decrease in the mechanical properties of the composite. Therefore, in the studied range of molding time, 60 min is selected.

**Figure 14:**
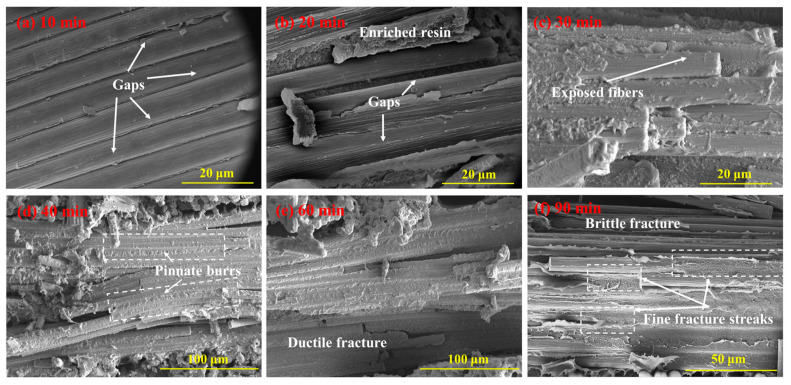
SEM images of typical fracture surfaces of CFF/PEEK composites by different molding times: (**a**) 10 min; (**b**) 20 min; (**c**) 30 min, (**d**) 40 min; (**e**) 60 min; and (**f**) 90 min.

The SEM images in Figure 14 illustrate the fracture surfaces under flexural loading of CFF/PEEK composites fabricated with different molding times. At a molding time of 10 min (Figure 14a), noticeable gaps can be observed between fibers due to insufficient time for PEEK resin infiltration into CFF. As the molding time increased to 20 min and 30 min (Figure 14b,c), the impregnation of the fiber surface improved, but exposed fibers and gaps still persisted. When molding time reaches 40 min, most fibers are covered by resin while feathery burrs appear on their surfaces, exhibiting pinnate burrs in localized areas and indicating plastic deformation and ductile fracture behavior of PEEK resin (Figure 14d). For the case of 60 min holding time, fiber surfaces become more evenly coated with resin while distinct feathery burrs are observed in cross-sections indicating typical plastic fracture mode occurrence, demonstrating good impregnation degree and strong fiber–matrix interface strength. However, as the molding time reached 90 min, fine fracture streaks appeared on the sample’s fracture surface while plastic deformation disappeared (Figure 14e), indicating typical characteristics of brittle fracture. This change in fracturing morphology can be attributed to excessive crosslinking density as a result of prolonged molding time. Optimal molding time ranging from 40–60 min ensure full infiltration of CFF by PEEK resin without excessive crosslinking, promoting a strong bond between fiber and matrix and thereby enhancing mechanical properties of CFF/PEEK composites. However, an excessively prolonged molding time can lead to the excessive crosslinking of PEEK resin, resulting in a transition from toughness to brittleness and even slight thermal degradation. As a consequence, this weakens the fiber–matrix bonding strength and leads to a decrease in mechanical properties.

### 3.5. The Effect of Pre-Compaction Process on the Internal Structure of CFF/PEEK Composite

**Figure 15:**
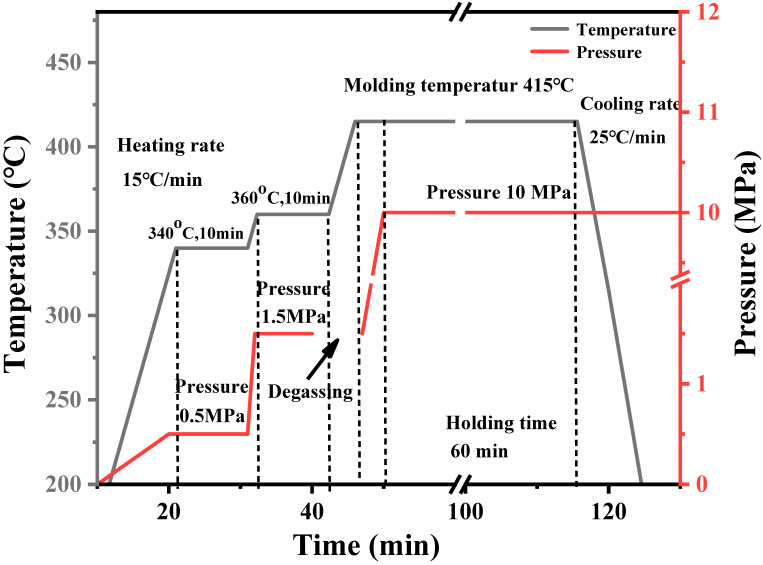
Molding procedure of CFF/PEEK composite with pre-pressure.

The effect of the pre-compaction process on the composite properties is noteworthy. During the processing of the composite, applying a certain pressure below the molding temperature to pre-compact the resin matrix and reinforcement materials can help discharge gas before the resin melts, thereby reducing the defect of the product. In this work, a pressure of 0.5 MPa was applied at 340 °C for 10 min, then the temperature was increased to 360 °C, and a pressure of 1.5 MPa was applied for 10 min. After pre-pressing, the temperature was increased to 415 °C, and a pressure of 10 MPa was maintained for 60 min. The hot-pressing process is shown in Figure 15.

**Figure 16:**
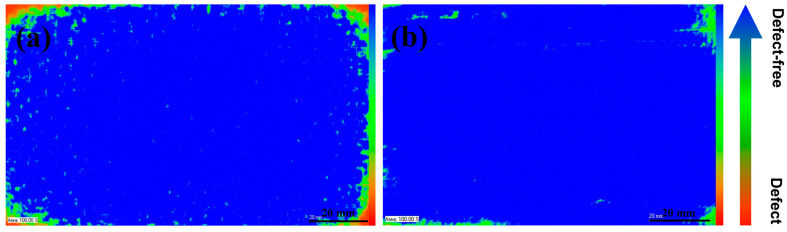
C-SAM images of CFF/PEEK composites with pre-pressure: (**a**) without pre-pressure and (**b**) with pre-pressure.

Figure 16a shows a C-SAM image of the composite without the pre-compaction process, and Figure 16b shows a C-SAM image of the composite with the pre-compaction process. It can be clearly seen from the pictures that the defective areas of the composite with pre-compaction have significantly decreased, and the compacted area inside the lamellar has increased, especially at the edges of the composite. This may because of that during the hot-pressing process of composite, pre-compaction softens the PEEK film and allows it to infiltrate into the CFF under a certain pressure. During the degassing stage, it is easier to discharge gas bubbles under a certain pressure. Then, at the hot-pressing temperature, the PEEK film melts, and pressure is increased, making it easier for the melt to fully infiltrate into the CFF, thereby avoiding internal defects such as bubbles and delamination in the composite.

**Figure 17:**
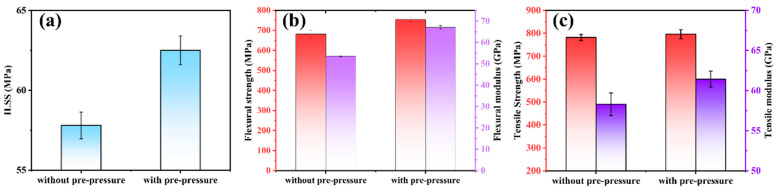
Relationships between mechanical properties and with or without pre-pressure of CFF/PEEK composites: (**a**) ILSS; (**b**) flexural strength and flexural modulus; and (**c**) tensile strength and tensile modulus.

Table 4 shows the test results of the mechanical properties of the CFF/PEEK composite with and without the pre-compaction process. Figure 17 gives a comparative histogram of the mechanical properties of the corresponding composites. After the pre-compaction process, the interlaminar strength of the composite material increased from 57.8 MPa to 62.5 MPa, and the flexural strength increased from 681.5 MPa to 754.4 MPa, but there was no significant change in the tensile strength. In the tensile direction, the tensile strength is mainly determined by the performance of the fiber reinforcement. While the shear strength, compared with the tensile strength, depends more on the interface bonding strength between the two phases of the material. Thus, the pre-compaction process was used for preparing CFF/PEEK composites.

**Figure 18:**
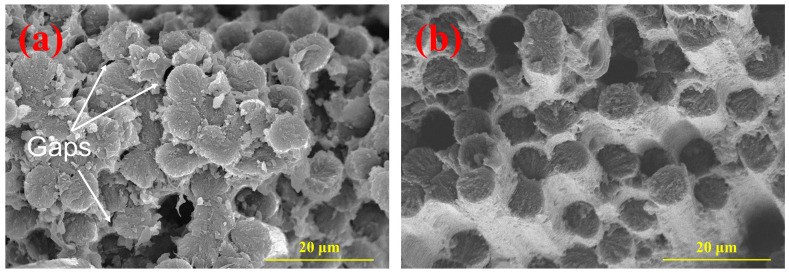
SEM images of typical fracture surfaces of CFF/PEEK composites: (**a**) without pre-pressure and (**b**) with pre-pressure.

By optimizing the molding process parameters, such as molding temperature, molding pressure, and mold time, the PEEK resin can effectively infiltrate the face of the CFF, resulting in enhanced mechanical properties of the composite material. However, some defects can be observed around the composite plate through ultrasonic scanning microscope images, leading to reduced product utilization rates. Additionally, small gaps between fibers were found in the SEM image (Figure 18a), which cannot be detected by ultrasonic scanning microscopy due to their relatively small size. These gaps can be attributed to the trapped air between fibers. Although most of the air can be expelled under higher pressure, it is difficult to discharge air between individual fibers within a tow. The pre-compaction process adds pressure before the complete melting or flow of PEEK resin, which can facilitate the expulsion of air between and within fibers. Subsequently, heating and pressurization are employed to further eliminate air trapped between individual fibers in the tow, enabling rapid high-temperature encapsulation by a resin matrix with excellent fluidity. This approach effectively reduces product defect rates (Figure 18b) while promoting improved utilization through comprehensive removal of air from the composite material system.

## 4. Discussion

Here, the relationships between process, structure, and property of plain-woven CFF/PEEK thermoplastic composites were studied. CFF/PEEK thermoplastic composites were fabricated using a simple film stacking method with the hot-pressing process. The film stacking method of PEEK film and CFF can solve the difficulty of extending molten PEEK in the plane direction and make PEEK resin evenly distributed between each layer of CFF, which could efficiently avoid defects in the composite material. Four key process parameters, including molding temperature (370, 385, 400, and 415 °C), molding pressure (1, 2, 4, 8, and 10 MPa), molding time (10, 20, 30, 40, 60, and 90 min), and use of the pre-compaction process, and their effects on the mechanical properties of CFF/PEEK composites were explored.

When the temperature increases from 370 °C to 415 °C, there is a significant reduction in the melt viscosity of PEEK (Figure 2d). The reduction in viscosity of PEEK allows the molten resin to readily migrate into the interlayer and fiber bundles, thereby enhancing interlayer adhesion and fiber/resin interaction. This migration of resin into the fiber bundles and surrounding areas facilitates a decrease in void defects inside the composite material (Figure 4). Additionally, improved fluidity promotes a more uniform distribution of resin, resulting in enhanced force transfer efficiency and stronger interlayer adhesion (Figure 6). These factors collectively contribute to improved mechanical properties of CFF/PEEK composites. Based on these reasons, when the molding temperature was 415 °C, the ILSS of the composite material increased by 43% from 40.5 MPa to 57.8 MPa. The flexural strength increased by 42%, from 455.9 MPa to 681.5 MPa, while the tensile strength increased by 20%, from 652.2 MPa to 781.8 MPa.

As the molding pressure increased from 1 to 10 MPa, the melted PEEK was pressed into the fibers, improving fiber/resin impregnation and reducing defects in the composite (Figure 8). This enhanced the deformation resistance of the CFF/PEEK composite, which enhanced the force transmission ability between fibers and matrix. The lower molding pressure was not enough to allow the high-viscosity PEEK resin to flow and penetrate between the fibers, and the entrapped air inside was difficult to completely remove, which led to many defects inside the composite, thereby reducing the mechanical properties. When the pressure reached an optimal level, the intensified pressure made the high-viscosity resin infiltrate into the CFF while discharging the residual air inside, thereby reducing the porosity inside the composite material and significantly improving its mechanical properties. When the molding pressure was 10 MPa, the molten PEEK resin could impregnate the fiber very well and improve the mechanical properties of CFF/PEEK composites (Figure 10). The ILSS, flexural strength, and tensile strength were 57.8 MPa, 681.5 MPa, and 781.8 MPa, respectively.

The PEEK resin possesses a high viscosity and a correspondingly slow flow rate. Moreover, the densely packed fibers create an inherent barrier to the resin’s flow. Consequently, the molten PEEK resin requires sufficient time for thorough impregnation of the fiber bundles. However, an excessively prolonged molding time will cause a thermal crosslinking reaction within the PEEK in a high-temperature environment, significantly impacting the resin’s mechanical characteristics [33,34]. More critically, over-crosslinking may render the PEEK resin brittle and cause degradation. When the molding time increased from 10 to 60 min, the resin infiltrated into the interior of the CFF strands gradually. Meanwhile, the composite became more compact, and the area of defects significantly decreased (Figure 12). Obviously, at a molding time of 60 min, the fracture surface of the corresponding specimen was observed, revealing fibers fully encapsulated by PEEK resin. The PEEK resin had undergone plastic deformation as pinnate burrs and cusps appeared, indicative of ductile fracture characteristics, which signified a good impregnation degree and strong fiber–matrix interface strength (Figure 16). However, with further increases in holding time to 90 min, the emergence of fine fracture streaks was observed while plastic deformation disappeared, indicating typical characteristics of brittle fracture. This change in fracturing morphology can be attributed to excessive crosslinking density resulting from prolonged molding time. The optimal molding time of 60 min ensured complete infiltration of CFF by PEEK resin without excessive crosslinking, promoting a strong bond between fiber and matrix and thereby enhancing the mechanical properties of CFF/PEEK composites. At 60 min molding time, the mechanical properties reached their maximum values with a ILSS of 57.8 MPa, a flexural strength of 681.5 MPa, and a tensile strength of 781.8 MPa.

In the process of composites hot-pressing, the pre-compacted process between the resin matrix and reinforcement material is necessary. Below the molding temperature, most of the air between the bundles was discharged under pressure. Then, the temperature was raised to reach the melting point of the PEEK film. Increasing pressure facilitates full infiltration of molten PEEK into CFF, further eliminating air between fiber bundles. This allows for quick wrapping of high-temperature resin matrix with good fluidity around fibers, reducing product defect rate. Additionally, it ensures the thorough discharge of air within the composite material system, minimizing the defect rate around the composite. Moreover, it enhances the interface relationship between resin and fiber by promoting a stronger interlayer bonding force that enables the transfer of external stress from resin to fiber. Thus, the mechanical properties of composite material improve. After using the pre-compacted process, ILSS increased from 57.8 MPa to 62.5 MPa, while flexural strength rose from 681.5 MPa to 754.4 MPa and tensile strength was 796.l MPa.

Compared with previous works [13,14,21], the 1K CFF with a smaller bundle was employed in this work, which has received less attention regarding its composite material properties and processing. A smaller bundle of CFF indicates a tighter weave structure with reduced gaps between fibers, making it more challenging for the resin to fully and evenly infiltrate the surface of each carbon fiber. Consequently, the preparation process becomes more challenging. Additionally, an ultrasonic scanning microscope was used to accurately analyze internal pore and void defects within the composite material non-destructively, providing a comprehensive assessment of sample properties. When PEEK exhibits good infiltration on the CFF surface, there are fewer internal defects present in the material. Finally, under optimal hot-press processes, CFF/PEEK composites exhibit minimal defects while demonstrating enhanced resin-fiber bundle interaction and superior mechanical properties.

## 5. Conclusions

In this work, the relationships between the process, structure, and properties of CFF/PEEK composites were studied. Using the hot-pressing process and plain-woven CFF and PEEK films as materials, CFF/PEEK composites were fabricated using the film stacking method. Four key process parameters, including molding temperature, molding pressure, molding time, and pre-compaction process, were optimized, and their effects on the mechanical properties of CFF/PEEK composites were explored. Additionally, the influence mechanism of these process parameters was exposed. At lower molding temperatures, the viscosity of PEEK resin is higher, which results in poor impregnation of CFF/PEEK composites. The mechanical properties of CFF/PEEK composites increase with the increase of molding temperature. Under insufficient molding pressure, PEEK resin cannot efficiently penetrate into the CFF, resulting in weak fiber–matrix bonding strength. The mechanical properties of composites increase with the increase of molding pressure. Insufficient molding time cannot make molten PEEK resin fully impregnate the CFF. Sufficient molding time can ensure a good impregnation degree without damaging the toughness of PEEK resin. An overlong molding time produces excessive crosslinking of PEEK, leading to the brittleness of PEEK and weakening of the mechanical properties of CFF/PEEK composites. Using the pre-compaction process can reduce the tiny defects of the composites and make the stress transfer from the resin to the fiber. The mechanical properties of composites improve. Thus, the ideal process parameters are a molding temperature of 415 °C, molding pressure of 10 MPa, molding time of 60 min, and the use of the pre-compaction process. This research provides technical reference for optimizing 1K CFF-reinforced resin composite’s production systematically by revealing internal influence mechanisms on mechanical properties, demonstrating a new direction for achieving high-quality production of 1K CFF-reinforced resin composites. Further investigation will focus on the wettability of 1K CFF and PEEK fibers, the modification of CFF/PEEK, and the application of 1K CFF/PEEK composites.

## Figures and Tables

**Table 1:** Mechanical properties of CFF/PEEK composites with different molding temperatures.

Molding Temperature (°C)	ILSS (MPa)	Flexural Strength (MPa)	Flexural Modulus (GPa)	Tensile Strength (MPa)	Tensile Modulus (GPa)
370	40.5	455.9	64.6	652.2	57.2
385	47.3	509.9	65.7	738.8	58.4
400	51.2	601.8	65.9	752.7	59.4
415	57.8	681.5	66.3	781.8	58.3

**Table 2:** Mechanical properties of CFF/PEEK composites with different molding pressures.

Molding Press (Mpa)	ILSS (MPa)	Flexural Strength (MPa)	Flexural Modulus (GPa)	Tensile Strength (MPa)	Tensile Modulus (GPa)
1	42.4	478.7	63	662.1	53.9
2	50.9	561.2	64.5	673.6	57.6
4	52.9	601.8	65.4	716.2	57.7
8	56	641.6	66.1	753.9	58.2
10	57.8	681.5	66.3	781.8	58.3

**Table 3:** Mechanical properties of CFF/PEEK composites with different molding times.

Molding Time (min)	ILSS (MPa)	Flexural Strength (MPa)	Flexural Modulus (GPa)	Tensile Strength (MPa)	Tensile Modulus (GPa)
10	41.1	444.6	64.2	661.5	55.8
20	42.8	454.5	64.5	673.5	56.4
30	54.5	565.2	64.6	759.7	57.4
40	55.7	634.4	64.6	762.7	57.8
60	57.8	681.5	66.3	781.8	58.3
90	55.5	626.4	65.1	745.8	57.5

**Table 4:** Mechanical properties of CFF/PEEK composites with or without pre-pressure.

	ILSS (MPa)	Flexural Strength (MPa)	Flexural Modulus (GPa)	Tensile Strength (MPa)	Tensile Modulus (GPa)
Without pre-pressure	57.8	681.5	53.6	781.8	58.3
With pre-pressure	62.5	754.4	67.1	796.1	61.4

## Data Availability

Data are contained within the article.
