# Correction: Wang et al. Study on the Preparation and Process Parameter-Mechanical Property Relationships of Carbon Fiber Fabric Reinforced Poly(Ether Ether Ketone) Thermoplastic Composites. *Polymers* 2024, *16*, 897

**DOI:** 10.3390/polym18091054

**Published:** 2026-04-27

**Authors:** Yan Wang, Yanchao Yang, Hongbo Zhang, Siwen Ding, Ting Yang, Jinhui Pang, Haibo Zhang, Jinling Zhang, Yunhe Zhang, Zhenhua Jiang

**Affiliations:** Key Laboratory of High Performance Plastics, Ministry of Education, National & Local Joint Engineering Laboratory for Synthesis Technology of High Performance Polymers, College of Chemistry, Jilin University, Changchun 130012, China; wyan2012@jlu.edu.cn (Y.W.); yangyanchao@jlu.edu.cn (Y.Y.); zhanghb20@mails.jlu.edu.cn (H.Z.); dingsw23@mails.jlu.edu.cn (S.D.); yangting1988@jlu.edu.cn (T.Y.); pangjinhui@jlu.edu.cn (J.P.); zhanghaib@jlu.edu.cn (H.Z.); jiangzhenhua@jlu.edu.cn (Z.J.)

**Error in Figure**

In the original publication [1], there was a mistake in Figure 10d. Due to an oversight by the author, incorrect image in Figure 10d was used. The corrected Figure 10 appears below. 

**Text Correction**

There was an error in the original publication. “Owing to the author’s oversight, the temperature value of 415 °C stated in the Conclusion and Abstract section were erroneously recorded as 410 °C”.

A correction has been made to “Abstract and Conclusions Section”:

“Abstract: The ultimate process parameters are a molding temperature of 415 °C, molding pressure of 10 MPa, molding time of 60 min, and the need for the pre-compaction process.”.

“Conclusions: Thus, the ideal process parameters are a molding temperature of 415 °C, molding pressure of 10 MPa, molding time of 60 min, and the use of the pre-compaction process.”.

The authors state that the scientific conclusions are unaffected. This correction was approved by the Academic Editor. The original publication has also been updated.

## Figures and Tables

**Figure 10:**
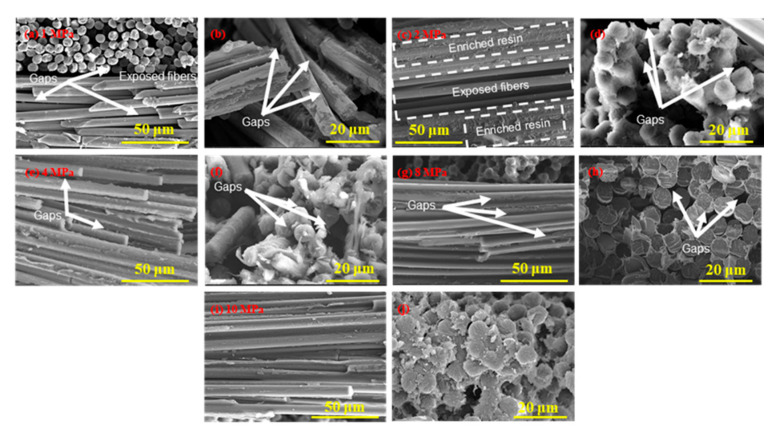
SEM images of typical fracture surfaces of CFF/PEEK composites by different molding pressures: (**a**,**b**) 1 MPa; (**c**,**d**) 2 MPa; (**e**,**f**) 4 MPa; (**g**,**h**) 8 Mpa; and (**i**,**j**) 10 MPa.
